# Subclinical Alterations of Cardiac Mechanics Present Early in the Course of Pediatric Type 1 Diabetes Mellitus: A Prospective Blinded Speckle Tracking Stress Echocardiography Study

**DOI:** 10.1155/2016/2583747

**Published:** 2015-12-29

**Authors:** Kai O. Hensel, Franziska Grimmer, Markus Roskopf, Andreas C. Jenke, Stefan Wirth, Andreas Heusch

**Affiliations:** Department of Pediatrics, HELIOS Medical Center Wuppertal, Centre for Clinical & Translational Research (CCTR), Centre for Biomedical Education & Research (ZBAF), Faculty of Health, Witten/Herdecke University, Heusnerstraße 40, 42283 Wuppertal, Germany

## Abstract

Diabetic cardiomyopathy substantially accounts for mortality in diabetes mellitus. The pathophysiological mechanism underlying diabetes-associated nonischemic heart failure is poorly understood and clinical data on myocardial mechanics in early stages of diabetes are lacking. In this study we utilize speckle tracking echocardiography combined with physical stress testing in order to evaluate whether left ventricular (LV) myocardial performance is altered early in the course of uncomplicated type 1 diabetes mellitus (T1DM). 40 consecutive asymptomatic normotensive children and adolescents with T1DM (mean age 11.5 ± 3.1 years and mean disease duration 4.3 ± 3.5 years) and 44 age- and gender-matched healthy controls were assessed using conventional and quantitative echocardiography (strain and strain rate) during bicycle ergometer stress testing. Strikingly, T1DM patients had increased LV longitudinal (*p* = 0.019) and circumferential (*p* = 0.016) strain rate both at rest and during exercise (*p* = 0.021). This was more pronounced in T1DM patients with a longer disease duration (*p* = 0.038). T1DM patients with serum HbA_1c_ > 9% showed impaired longitudinal (*p* = 0.008) and circumferential strain (*p* = 0.005) and a reduced E/A-ratio (*p* = 0.018). In conclusion, asymptomatic T1DM patients have signs of hyperdynamic LV contractility early in the course of the disease. Moreover, poor glycemic control is associated with early subclinical LV systolic and diastolic impairment.

## 1. Introduction

Type 1 diabetes mellitus (T1DM) is ranging among the most common chronic disorders of childhood and adolescence [1] with increasing incidence worldwide [2, 3]. Cardiovascular disease is the most common cause of death in diabetic patients and currently one of the leading causes of death overall in the industrialized world [4]. While ischemic events range highest in the list of diabetic cardiovascular complications [5], diabetic patients also develop heart failure in the absence of arterial hypertension and myocardial ischemia [6–8]. Even though the existence of “diabetic cardiomyopathy” in humans is a current matter of ongoing scientific controversy [9, 10], there is growing evidence for the assumption that diabetes can lead to systolic and diastolic cardiac dysfunction without other obvious causes for cardiomyopathy, such as overt ischemia, coronary artery disease, arterial hypertension, or valvular heart disease [11–16]. While there is a variety of causes contributing to diabetes-associated heart failure including impaired calcium homeostasis, enhanced fatty acid metabolism, suppressed glucose oxidation, altered intracellular signaling, and pathologic remodeling, the underlying pathophysiology of diabetic cardiomyopathy is still not well understood [8]. Whether uncomplicated diabetes mellitus already affects myocardial function in asymptomatic children at an early stage of the disease currently remains elusive [17]. Hence, children and adolescents with uncomplicated diabetes may serve as an ideal model to study the effect of diabetic metabolic conditions in the absence of potentially confounding ischemic events.

Myocardial deformation is a complex three-dimensional process influenced by heterogeneously organized heart muscle fibers. Measurements of left ventricular (LV) function are important for the evaluation, management, and estimation of prognosis in patients with various forms of cardiovascular disease [18]. However, ejection fraction (EF), the current echocardiographic gold standard for the assessment of systolic function, bears considerable limitations as a prognostic parameter [19] and does not correlate well with quantitative measures of functional capacity [20]. It uses a simplistic approach based on visual assessment of inward motion and wall thickening that underestimates the true complexity of myocardial contraction and suffers from significant inter- and intrarater variability [21]. Thus, subtle alterations in myocardial wall motion remain occult. Speckle tracking echocardiography (STE) is a quantitative diagnostic method for the assessment of myocardial deformation [22]. STE derived measurements correlate well with functional capacity [23] and feature promising inter- and intraobserver reproducibility [24]. Moreover, STE has been shown to detect subclinical systolic LV impairment in asymptomatic patients with preserved EF and arterial hypertension [25] or heart failure [26], respectively.

The aim of this study was to investigate whether STE can be used to detect subclinical alterations of LV myocardial deformation in asymptomatic pediatric patients with uncomplicated T1DM. Furthermore, we combined STE with physical stress testing in order to unmask subtle changes of cardiac contractility that might potentially be occult at rest.

## 2. Methods

### 2.1. Study Population

For this prospective diagnostic study we enrolled 40 consecutive children and adolescents with T1DM aged 6 to 17 years (mean age 11.5 ± 3.1 years; 40% female) and 44 age- and sex-matched healthy controls (mean age 11.4 ± 2.9 years; 45% female). Mandatory inclusion criteria in the study group were the diagnosis of insulin-dependent T1DM and a good general health state. Exclusion criteria were other past or present medical conditions that may affect the cardiovascular system such as congenital heart disease, systemic inflammatory disease, for example, history of Kawasaki disease, proteinuria, the use of any type of systemically acting medication (other than insulin for the study group), developmental delay, body mass index > 30 kg/m^2^, submaximal effort during exercise testing, short leg length, or pathologic EKG-changes at rest or during exercise. None of the included patients suffered from sings of end-organ damage such as evidence of renal failure or retinal changes. Healthy control subjects had an entirely negative medical history with regard to the cardiovascular as well as to any other organ system. A written informed consent was obtained from each participant as well as from their legal guardian prior to inclusion in the study. Subsequently, a thorough history and physical examination as well as both resting and exercise echocardiography and EKG were obtained. The sample size was achieved by including all patients from the hospital's diabetes clinic that were willing to participate in the study. A priori study design was established dividing the diabetes population into subgroups of patients with a disease duration of less than 4 years (*n* = 23, 57.5%) and more than 4 years (*n* = 17, 42.5%) as well as a three-column stratification according to glycemic control with serum HbA_1c_ < 7.5% (*n* = 10, 25%), HbA_1c_ 7.5–9% (*n* = 19, 47.5%), and HbA_1c_ > 9% (*n* = 11, 27.5%). The study was approved by the Witten/Herdecke University ethics committee and carried out in accordance with declaration of Helsinki's ethical principles for medical research involving human subjects. The study was registered to the Witten/Herdecke University Ethics and Clinical Trials Committee and assigned the trial number 113/2013.

### 2.2. Conventional and Doppler Echocardiography

All examinations were performed with the commercially available ultrasound device iE33 by Phillips Ultrasound Inc., USA, using a S5-1 Sector Array transducer (Sector 1–5 MHz). All images were digitally recorded and transferred to an offline workstation for analysis, using XCelera Version 3.1.1.422 by Phillips Ultrasound Inc., USA. According to echocardiography guidelines a complete standard 2D study, as well as a spectral and color flow Doppler examination, was carried out [27]. Image acquisition was performed in the parasternal long axis view, three short axis views, and the apical 4-, 3-, and 2-chamber views. M-mode images were taken at level of the aortic valve and the LV for subsequent measurement of aortic root diameter, left atrial diameter, interventricular septum, LV cavity, and LV posterior wall. Fractional shortening, LV mass, relative wall thickness, LV end-diastolic/end-systolic volume, EF, stroke volume, and cardiac output were calculated. Utilizing pw-Doppler and pw-TDI E/A-ratio, E/E′-ratio, mitral deceleration time, and isovolumetric relaxation time were measured for the assessment of LV diastolic function as previously described [28]. All measurements were evaluated using *Z*-scores [29]. During the entire examination a particular focus was set on the exclusion of any congenital heart disease as well as morphological or functional abnormalities.

### 2.3. Speckle Tracking Echocardiography

Myocardial deformation parameters (strain and strain rate) were measured acquiring standard 2D grayscale LV images. Circumferential strain (CS) was assessed in the standard parasternal short axis at the mitral valve plane (SAXB) and the papillary muscle plane (SAXM). Longitudinal strain (LS) was measured with standard apical 4-chamber (AP4), 3-chamber (AP3), and 2-chamber (AP2) apical views using conventional B-Mode imaging as previously described [22]. Five consecutive heart beats synchronized to a continuous EKG were recorded with frame rate set between 60 and 90 frames per second as recently suggested [30]. Caution was paid to minimize artifacts and to reduce noise for most accurate 2D strain estimation. All loops were digitally stored anonymized in the DICOM format and transferred to an offline workstation for postprocessing using the commercially available software Qlab 9. Segmental and global LS and CS were measured in 7 and 6 segments per view, respectively, by manual tracing of the endocardial contour at end-systole. The following frames were automatically analyzed by temporal tracking of acoustic speckles that are individual to each segment of the myocardial tissue. Real-time verification of adequate tracking and full thickness coverage of the myocardium including the epicardial and endocardial borders were optimized by manual readjustment of poorly tracked segments where necessary. More negative strain and strain rate values will be described as “higher” in this paper, even though mathematically it is vice versa, as more negative values represent an increased contraction of the myocardium.

Both resting and exercise echocardiographic images were additionally analyzed by a second, independent reader who was blinded to the results of the first examiner and the study group status of the respective echocardiographic image in order to determine interobserver reliability.

### 2.4. Quantitative Stress Echocardiography

After the general echocardiographic studies, participants pedaled in a supine position utilizing a standard cycle ergometer at approximately 60 rounds per minute against a ramp protocol with increasing resistance. Image acquisition for speckle tracking deformation analyses was carried out in the resting state and at the maximum level of physical exhaustion (≈2 Watt per kilogram body weight). A standardized pattern of consecutive images was acquired at each time point in the following order: SAXB, SAXM, AP4, AP2, and AP3. A 12-channel EKG was continuously monitored and blood pressure measurements were collected at 2-minute intervals.

All echocardiographic analyses were performed by the same investigators, who were blinded to the study group status at the time of the assessment of strain and strain rate. The results were reproducible and interobserver variability was below 5.8% in our study.

In order to reduce the risk of exercise induced hypoglycemia in diabetic patients, serum glucose levels should exceed 100 mg/dL to 150 mg/dL. Patients with blood sugar levels below 100 mg/dL were provided with extra carbohydrate exchange such as candy bars or orange juice prior to physical exercise testing.

### 2.5. Biostatistical Analysis

Baseline demographics, clinical data, hemodynamic parameters, and echocardiographic characteristics of the two groups were described by mean and standard deviation. Clinical parameters, hemodynamic data, and echocardiographic characteristics of the two study groups were compared using the Mann-Whitney *U* test. Wilcoxon signed-rank test was used for the measurement of the effect of exercise within one group. *p* values <0.05 constituted statistical significance. Box-Whisker-Plots were used for the graphic representation of the data distribution. SPSS Statistics for Macintosh, Version 22.0. (IBM Corporation, USA), was used for all statistical analyses.

## 3. Results

### 3.1. Epidemiological Data

Baseline demographic and hemodynamic data of the study population are summarized in Table 1. There was no significant difference in age, body weight, height, bmi, or the level of exercise routine between the two groups. Blood pressure and heart rate did not differ between the two groups at rest or during stress testing except for a slightly higher heart rate in T1DM patients at rest (84.4 ± 11.3 bpm) when compared to healthy controls (76.2 ± 9.3 bpm; *p* = 0.001). However, all baseline and hemodynamic parameters in both groups were within normal limits [31]. Mean disease duration in the study group was 4 ± 3.5 years and mean glycated hemoglobin (HbA_1c_) was 8.3 ± 1.2%. One patient was excluded from the study due to detection of a previously unknown valvular aortic stenosis.

### 3.2. Conventional Echocardiography

Conventional echocardiographic characteristics are outlined in Table 2. There are no significant differences of atrial/aortic diameters or LV function parameters such as fractional shortening, EF, stroke volume, and cardiac output, except for a marginally larger systolic diameter of the interventricular septum in the control group (1.17 ± 0.20 cm) when compared to T1DM patients (1.06 ± 0.22 cm). Yet all values were within the normal range evaluated by *Z*-scores [29]. Analysis of diastolic function showed a significantly decreased E/A-ratio in the T1DM group (1.6 ± 0.28) when compared to healthy controls (1.72 ± 0.26; *p* = 0.031). E/E′-ratio, IVRT, and mitral deceleration time as well as all other assessed parameters of diastolic function showed no significant differences between the two groups.

### 3.3. Speckle Tracking Stress Echocardiography

Myocardial deformation was quantitatively measured using speckle tracking echocardiography at rest and during physical stress testing on a bicycle ergometer. Results for peak LV myocardial* strain rate* are displayed in Table 3. T1DM patients were shown to have increased myocardial contractility both at rest (*p* = 0.016) and during stress (*p* = 0.021). While statistical significance was reached in 4 out of 14 comparisons, T1DM patients had higher circumferential and longitudinal* strain rate* than healthy controls in 13 out of 14 comparisons. The significance of the difference of AP2 derived longitudinal strain rate in the diabetic and control group at rest (−1.94 ± 1.14 versus −1.54 ± 0.25 s^−1^) is limited by the nonnormal distribution of the parameters.

**Figure 1:**
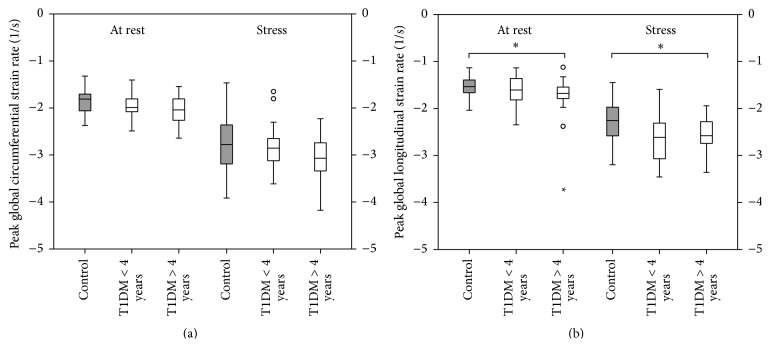
Peak systolic global left ventricular* strain rate* in relation to the duration of disease. Type 1 diabetic children (*n* = 40) have increased strain rate both at rest and during exercise when compared to healthy controls (*n* = 44). Patients with a disease duration >4 years (*n* = 17, 42.5%) exhibit higher strain rates than those with a disease duration <4 years (*n* = 23, 57.5%). (a) Peak systolic global LV circumferential* strain rate*. (b) Peak systolic global LV longitudinal* strain rate*. ^*∗*^ *p* < 0.05; *p* values were calculated with Mann-Whitney *U* and Wilcoxon signed-rank tests.

**Figure 2:**
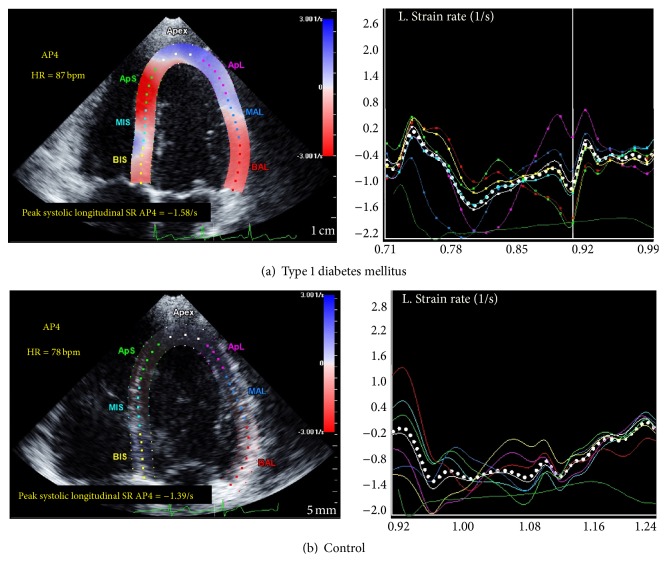
Speckle tracking echocardiography at rest in the apical 4-chamber view. (a) Peak systolic global LV longitudinal* strain rate* in a pediatric patient with type 1 diabetes mellitus. (b) Peak systolic global LV longitudinal* strain rate* in a healthy control subject. Dotted white line: global longitudinal* strain rate*, the coloured lines on the right correspond to the myocardial segments indicated on the left, dark green line at the bottom: ECG. Note the increased peak early systolic strain rate in the diabetic patient.

The effect of disease duration on LV myocardial contractility is demonstrated in Figure 1. While T1DM patients had overall higher global LV* strain rates* at rest when compared to healthy controls, patients with a disease duration of >4 years had significantly increased LV* strain rate* at rest (*p* = 0.038) and during exercise (*p* = 0.05).  Figure 2 illustrates generic speckle tracking echocardiography images of increased peak LV systolic* strain rate* in a patient with T1DM and a healthy sex- and age-matched control subject.

**Figure 3:**
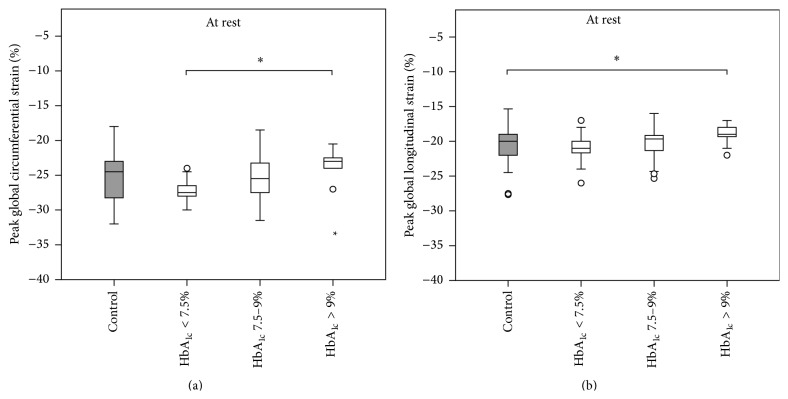
Peak systolic global left ventricular* strain* in relation to glycemic control. Type 1 diabetic children with poor glycemic control have decreased peak systolic global left ventricular* strain* when compared to healthy controls. (a) Peak systolic global LV circumferential* strain*. (b) Peak systolic global LV longitudinal* strain*. HbA_1c_ < 7.5% (*n* = 10, 25%), HbA_1c_ 7.5–9% (*n* = 19, 47.5%), and HbA_1c_ > 9% (*n* = 11, 27.5%); ^*∗*^ *p* < 0.05; *p* values were calculated with Mann-Whitney *U* and Wilcoxon signed-rank tests.

Overall, peak LV myocardial* strain* was not shown to be statistically different between the two groups, neither at rest, nor during physical exercise (see Table 4). However, significant differences could be demonstrated when analyzing the T1DM group stratified by glycemic control as visualized in Figure 3. T1DM patients with serum HbA_1c_ > 9% had significantly depressed peak LV CS (*p* = 0.005) and LS (*p* = 0.008) when compared to diabetic patients with better glycemic control and healthy controls, respectively.

**Figure 4:**
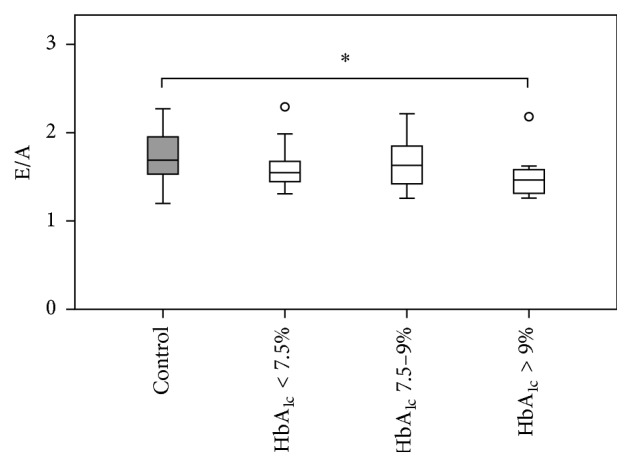
Left ventricular diastolic function (E/A-ratio) in relation to glycemic control. Type 1 diabetic children with poor glycemic control have echocardiographic evidence of impaired diastolic filling of the left ventricle in comparison to healthy control subjects.^*∗*^ *p* < 0.05; *p* values were calculated with Mann-Whitney *U* and Wilcoxon signed-rank tests.

T1DM patients showed beginning impairment of LV diastolic function (E/A-ratio) when compared to healthy controls (see Table 2). This effect was statistically significant for the comparison of the entire T1DM group to healthy controls (*p* = 0.031) and more pronounced in patients with poor glycemic control represented by serum HbA_1c_ levels > 9% (*p* = 0.018) as visualized in Figure 4.

## 4. Discussion

### 4.1. Type 1 Diabetic Children Have Increased Peak Left Ventricular Strain Rate

In order to assess the effect of type 1 diabetes mellitus on LV myocardial contractility in the absence of ischemic events early in the course of the disease we performed speckle tracking echocardiography in combination with ergometer stress testing in asymptomatic normotensive pediatric patients with uncomplicated T1DM and healthy controls. Interestingly and somewhat counterintuitively, we found diabetic children to exhibit LV systolic hypercontractility represented by overall increased peak circumferential and longitudinal* strain rate* both at rest and during exercise (see Table 3 and Figures 1 and 2). The observed statistically significant increases in LV strain rate in the T1DM group such as increased global longitudinal strain rate during stress testing (−2.59 ± 0.47 versus −2.32 ± 0.41 s^−1^) should not be overinterpreted as a single finding with direct clinical implication but rather regarded as the tip of the iceberg of the overall tendency for T1DM patients to exhibit increased peak systolic LV strain rate. At first, this may seem surprising given the fact that diabetic cardiomyopathy potentially results in a gradual decline of myocardial function with the ultimate end-point of diabetic heart failure. However, we hypothesize that diabetic cardiomyopathy may in fact feature an early subclinical phase of paradoxical LV hyperdynamics as a sign of impaired mechanical efficiency long before long-term deterioration of myocardial function becomes evident. While most studies focus on intermediate or late stage disease reporting of depressed LV systolic function, there are a number of human and animal model studies in favor of our hypothesis.

Chung et al. found increased LV torsion despite preserved EF, circumferential strain, and longitudinal shortening using tagged MRI in young adult patients with tightly controlled T1DM [32]. Similarly, a stress MRI spectroscopy study revealed a reduced phosphocreatine/*γ*-ATP ratio as a sign of altered myocardial energetics in young adults with uncomplicated T1DM, independent of coronary microvascular function [33]. In another tagged MRI study hyperdynamic LV twist mechanics were described in coexistence with signs of altered myocardial perfusion in young patients with uncomplicated T1DM [34]. Moreover, our results are in accordance with two conventional echocardiographic studies demonstrating increased LV contractility in diabetic children without arterial hypertension, ischemic heart disease, or nephropathy using M-mode and Doppler imaging [35, 36]. Furthermore, our finding of LV hyperdynamic contractility early in the course of diabetes mellitus is in agreement with results from animal model studies in leptin receptor-deficient mice utilizing in vivo catheterization. Buchanan et al. discovered diabetes-associated LV hypercontractility as an indication for altered myocardial substrate use and reduced myocardial efficiency in hyperglycemia. The phenomenon occurred early and slightly faded subsequently [37]. Additionally, Van den Bergh et al. described impaired mechanical efficiency and increased ventriculoarterial coupling that was associated with altered cardiac loading conditions [38]. Therefore, for the assessment of myocardial contractility in human subjects, a noninvasive measure that is least dependent on variations in LV loading must be utilized in order to minimize potential confounding.

Strain and strain rate are quantitative measures for the echocardiographic assessment of myocardial deformation [39]. Strain is an index of deformation describing a percentage change from the original dimension. Strain rate is a measure of the rate at which this change happens and is expressed as per second (s^−1^). While most studies assessing myocardial deformation in diabetic patients mainly focus on strain, strain rate is in fact a more robust index of LV myocardial contractility as it is less dependent on confounding factors such as pre- and afterload [40–43] and it is even more closely related to contractility than the widely used EF [44]. The present study is the first clinical study demonstrating overall increased strain rate in the early stage of human T1DM.

In contrast, Di Cori and colleagues used tissue-Doppler imaging to analyze myocardial deformation in adult T1DM patients (mean age 30 ± 4.1 years and mean disease duration 8.9 ± 3.7 years) and found depressed LV myocardial strain and equivocal strain rate [45]. There are several explanations for this deviation in deformation parameters. First, Di Cori and colleagues only assessed regional segmental strain (rate) of the midposterior septum (decreased in T1DM) and midlateral wall (increased in T1DM) and not global strain (rate). Given the strong heterogeneity of myocardial fiber organization in the LV, myocardial deformation naturally exhibits regional variations [46–48]. Thus, the assessment of only two isolated segments likely is an oversimplification of the complex mechanism underlying LV myocardial contractility. Second, tissue-Doppler echocardiography was used, a method that bears considerable limitations such as angle-dependency and interrater variability [49]. Third, Di Cori and colleagues included an older diabetic study population with a markedly longer disease duration in comparison to the present study population. In our study the mean disease duration in the T1DM group was 4 ± 3.5 years. Interestingly, a subgroup analysis revealed that the increase in global LV peak longitudinal strain rate is statistically significant for T1DM patients with a disease duration >4 years (see Figure 1). Hence, it is well imaginable that the here described diabetes-associated cardiac changes require a certain time interval of a few years to become evident. Furthermore, the observed hypercontractility in T1DM in the present study is possibly a transient effect in the early phase of diabetic nonischemic cardiomyopathy that fades in the subsequent course of the disease, as observed by Di Cori and colleagues. Longitudinal studies are needed in order to further elucidate the natural course of diabetic cardiomyopathy throughout childhood and adulthood.

### 4.2. Left Ventricular Longitudinal and Circumferential Strain Is Impaired in Children with Poorly Controlled Type 1 Diabetes Mellitus

In this study there was no significant difference in overall peak LV longitudinal or circumferential strain between T1DM patients and healthy controls neither at rest nor during stress testing. This is in accordance with a Korean study of a very similar (age, disease duration, and glycemic control) T1DM population that also failed to demonstrate overall impairment of systolic strain [50]. Strain rate however was not measured in that study. While there is a considerable number of clinical studies reporting an impairment of (mainly global longitudinal) strain in diabetes mellitus type 1 [51–53] and type 2 [54], all of these studies either include adult patients and/or are confounded by longer disease duration [51, 55–57], LV structural abnormalities [51, 55, 58], impaired EF [52], obesity, arterial hypertension [51, 54, 59–61], nephropathy [51, 57, 61], heart failure [55], overt peripheral vascular disease [56], use of negatively inotropic medications [51, 54, 60], or tobacco use [51]. Furthermore, in contrast to our study design all of the abovementioned studies are considerably limited by the fact that the echocardiographic interpreter was not blinded. This is a substantial limitation because speckle tracking derived myocardial deformation parameters are extremely sensible to manual adjustments.

Recently, a blinded speckle tracking study in 1065 normotensive T1DM patients (mean age 49.5 ± 14.5 years and mean disease duration 26.1 ± 15.7 years) convincingly demonstrated that the impairment of myocardial strain in T1DM is solely driven by the presence of albuminuria [62]. There was no difference in myocardial strain between T1DM patients without albuminuria and healthy controls. Strain rate however was not assessed in that study. As our study participants were screened negative for albuminuria and disease duration was considerably shorter than in the abovementioned study, the absence of overall impaired systolic strain in the present study population is not surprising. Moreover, our findings are in concordance with two MRI studies demonstrating preserved LV strain mechanics in young adult diabetic patients [63, 64].

Subdividing our T1DM population according to the degree of glycemic control, an association of both longitudinal and circumferential strains with serum levels of HbA_1c_ became evident (see Figure 3). This is in agreement with recent 3D speckle tracking studies demonstrating a negative impact of HbA_1c_ on LV myocardial strain in adult patients with diabetes mellitus [55, 65, 66]. Furthermore, this is underlined by prospective observational studies reporting the association of poor glycemic control with the development of heart failure in large cohorts of T1DM patients [5, 67]. In addition, several animal model studies are in accordance with our observations of diabetes-associated alterations in LV myocardial contractility [68, 69]. Accountable pathologic mechanisms are diabetes-induced loss of t-tubule structure [14], formation of advanced glycosylation end products with subsequent pathologically increased collagen cross-linking [70], altered mitochondrial energetics [71], and several other metabolic imbalances [11, 72]. The finding of overall preserved myocardial strain in the entire diabetic study population and decreased strain only in those subjects with poor glycemic control can be explained by the timing of the investigation. At this early state of T1DM only subjects with poor glycemic control exhibit advanced impairment of LV strain. The majority of the included patients either do not yet suffer from impaired contractility or are still in the previously described early occurring hyperdynamic state of diabetic cardiomyopathy. Taken together, our findings demonstrate the presence of early subclinical cardiac changes in diabetes mellitus that are most probably driven by metabolic dysfunction as previously suggested [73].

### 4.3. Type 1 Diabetic Children Have Signs of Beginning Diastolic Dysfunction

The present study provides further evidence for the presence of diastolic dysfunction in diabetes mellitus. We found a statistically significant decrease of E/A-ratio in poorly controlled T1DM patients when compared to healthy controls (see Figure 4). This is in accordance with observations in animal models [74–76] as well as with human MRI [64] and echocardiographic studies in pediatric [53] and adult [33, 77–80] patients with diabetes mellitus type 1 [33, 53, 80] and type 2 [77, 78] demonstrating signs of (beginning) diastolic dysfunction in nonischemic diabetic cardiomyopathy. A new aspect from the present study is the fact that signs of diastolic impairment already become evident very early in the course of T1DM. This further underlines the concept of nonischemic diabetes-associated myocardial impairment as a continuous process driven by metabolic imbalances.

### 4.4. Study Limitations

In a recent study on premature infants Sanchez and colleagues demonstrated a link of the reliability of two-dimensional speckle tracking derived deformation parameters and adjusted frame rate during image acquisition [81]. A frame rate/heart rate ratio of 0.7 to 0.9 frames per second per bpm has been proposed for optimal myocardial speckle tracking. In our study frame rate settings meet these criteria during echocardiography at rest. However, frame rates were not adjusted during stress testing. Accordingly, strain and strain rate parameters during exercise testing in our study may in fact be somewhat underestimated in both the study and the control group. Secondly, this was a cross-sectional study in a limited number of asymptomatic patients. Thus, the final clinical outcome of the observed subclinical alterations yet remains to be established in large study populations.

## 5. Conclusion

The present study provides further evidence for diabetes-associated nonischemic cardiomyopathy. A paradoxical increase of LV myocardial performance may occur very early in T1DM as a sign of impaired mechanical efficiency. T1DM patients with poor glycemic control have early signs of subclinical LV systolic and diastolic dysfunction. Consequently, tight glycemic control must be a high priority therapeutic aim for diabetic patients in order to minimize the ultimate risk of heart failure. Further experimental and clinical studies are needed in order to illuminate the spatiotemporal complexity of diabetes-associated heart failure.

## Figures and Tables

**Table 1:** Baseline clinical characteristics and hemodynamics of the study population.

	Diabetes (*n* = 40)	Control (*n* = 44)	*p* value
Age (years)	11.5 ± 3.1	11.4 ± 2.9	n.s.
Height (cm)	153.0 ± 18.2	154.1 ± 16.8	n.s.
Weight (kg)	46.9 ± 16.9	48.0 ± 16.3	n.s.
Body surface (m^2^)	1.4 ± 0.3	1.4 ± 0.3	n.s.
Body mass index (kg/m^2^)	19.3 ± 3.1	19.6 ± 3.5	n.s.
Exercise routine (1: in school; 2: <3 times/week; 3: ≥3 times/week)	1.7 ± 0.8	2.0 ± 0.7	n.s.
Duration of disease (years)	4 ± 3.5	—	—
HbA_1c_ (%)	8.3 ± 1.2	—	—
Rest			
Heart rate (beats/minute)	84.4 ± 11.3	76.2 ± 9.4	0.001
BP systolic (mmHg)	105.7 ± 9.6	105.8 ± 9.2	n.s.
BP diastolic (mmHg)	58.5 ± 7.9	59.4 ± 9.2	n.s.
Low stress level			
Heart rate (beats/minute)	112.0 ± 9.3	108.6 ± 12.8	n.s.
BP systolic (mmHg)	122.3 ± 18.2	120.2 ± 16.9	n.s.
BP diastolic (mmHg)	65.2 ± 13.6	63.7 ± 11.0	n.s.
Level of resistance (W/kg body weight)	0.4 ± 0.3	0.5 ± 0.3	n.s.
High stress level			
Heart rate (beats/minute)	161.5 ± 13.1	156.8 ± 17.5	n.s.
BP systolic (mmHg)	148.1 ± 21.9	140.1 ± 22.9	n.s.
BP diastolic (mmHg)	74.9 ± 12.6	71.2 ± 13.8	n.s.
Level of resistance (W/kg body weight)	1.7 ± 0.4	1.8 ± 0.4	n.s.

*p* values calculated with the Man-Whitney *U* test, level of significance = 0.05.

**Table 2:** Conventional echocardiographic parameters derived from two-dimensional and Doppler imaging.

	Diabetes (*n* = 40)	Control (*n* = 44)	*p* value
Aortic root (AoR) diameter (cm)	2.33 ± 0.35	2.41 ± 0.35	n.s.
Left atrial (LA) diameter (cm)	2.56 ± 0.38	2.71 ± 0.45	n.s.
LA/AoR	1.11 ± 0.16	1.13 ± 0.16	n.s.
Fractional shortening (%)	33.41 ± 4.08	34.78 ± 3.94	n.s.
Interventricular septal end-systolic diameter (cm)	1.06 ± 0.22	1.17 ± 0.20	0.011
Interventricular septal end-diastolic diameter (cm)	0.84 ± 0.18	0.89 ± 0.16	n.s.
LV end-systolic diameter (cm)	2.70 ± 0.43	2.76 ± 0.41	n.s.
LV end-diastolic diameter (cm)	4.05 ± 0.56	4.27 ± 0.46	n.s.
LV posterior wall diameter, systolic (cm)	1.23 ± 0.20	1.27 ± 0.21	n.s.
LV posterior wall diameter, diastolic (cm)	0.79 ± 0.16	0.81 ± 0.15	n.s.
LV mass (g)	102.74 ± 41.82	115.18 ± 37.56	n.s.
Relative wall thickness	0.20 ± 0.04	0.19 ± 0.03	n.s.
End-diastolic volume of the left ventricle (mL)	70.18 ± 24.66	79.63 ± 27.97	n.s.
End-systolic volume of the left ventricle (mL)	27.69 ± 9.82	31.66 ± 11.78	n.s.
Ejection fraction (%)	61.29 ± 4.77	60.16 ± 4.67	n.s.
Stroke volume (mL)	44.7 ± 14.4	49.3 ± 18.1	n.s.
Cardiac output (L/min)	3.7 ± 1.1	3.7 ± 1.3	n.s.
Mitral inflow: E-wave (cm/s)	95.36 ± 13.45	96.86 ± 14.26	n.s.
Mitral inflow: A-wave (cm/s)	60.84 ± 12.27	57.36 ± 10.41	n.s.
E-wave/A-wave	1.60 ± 0.28	1.72 ± 0.26	0.031
Mitral deceleration time (s)	0.17 ± 0.04	0.18 ± 0.04	n.s.
Isovolumetric relaxation time (s)	0.05 ± 0.01	0.05 ± 0.01	n.s.
S′ (cm/s)	7.92 ± 0.97	8.17 ± 1.19	n.s.
E′ (cm/s)	12.54 ± 1.81	13.03 ± 1.87	n.s.
A′ (cm/s)	5.42 ± 1.20	5.51 ± 1.11	n.s.
E′/A′ (cm/s)	2.44 ± 0.75	2.48 ± 0.72	n.s.
E/E′ (cm/s)	7.71 ± 1.20	7.56 ± 1.42	n.s.

*p* values calculated with the Man-Whitney *U* test, level of significance = 0.05.

**Table 3:** Speckle tracking derived peak systolic LV *strain rate* at rest and during stress testing.

	Diabetes (*n* = 40)	Control (*n* = 44)	*p* value
Rest			
Global circumferential strain rate (s^−1^)	−1.99 ± 0.28	−1.87 ± 0.24	n.s.
Circumferential strain rate (SAXM) (s^−1^)	−2.05 ± 0.35	−1.86 ± 0.25	0.016
Circumferential strain rate (SAXB) (s^−1^)	−1.96 ± 0.29	1.90 ± 0.31	n.s.
Global longitudinal strain rate (s^−1^)	−1.70 ± 0.44	−1.55 ± 0.21	n.s.
Longitudinal strain rate (AP4) (s^−1^)	−1.58 ± 0.34	−1.52 ± 0.28	n.s.
Longitudinal strain rate (AP2) (s^−1^)	−1.94 ± 1.14	−1.54 ± 0.25	0.019
Longitudinal strain rate (AP3) (s^−1^)	−1.64 ± 0.35	−1.63 ± 0.30	n.s.
Stress			
Global circumferential strain rate (s^−1^)	−2.92 ± 0.54	−2.76 ± 0.60	n.s.
Circumferential strain rate (SAXM) (s^−1^)	−2.92 ± 0.58	−2.73 ± 0.61	n.s.
Circumferential strain rate (SAXB) (s^−1^)	−2.86 ± 0.52	−2.66 ± 0.68	n.s.
Global longitudinal strain rate (s^−1^)	−2.59 ± 0.47	−2.32 ± 0.41	0.021
Longitudinal strain rate (AP4) (s^−1^)	−2.64 ± 0.53	−2.23 ± 0.37	0.002
Longitudinal strain rate (AP2) (s^−1^)	−2.40 ± 0.47	−2.47 ± 0.45	n.s.
Longitudinal strain rate (AP3) (s^−1^)	−2.71 ± 0.72	−2.60 ± 0.75	n.s.

SAXM: parasternal short axis view at the papillary muscle plane, SAXB: parasternal short axis view at the mitral valve plane, AP4: apical four-chamber view, AP2: apical two-chamber view, and AP3: apical three-chamber view; *p* values calculated with Man-Whitney *U* test, level of significance = 0.05.

**Table 4:** Speckle tracking derived peak systolic LV *strain* at rest and during stress echocardiography.

	Diabetes (*n* = 40)	Control (*n* = 44)	*p* value
Rest			
Global circumferential strain rate (%)	−25.5 ± 3.3	−25.0 ± 3.4	n.s.
Circumferential strain rate (SAXM) (%)	−26.6 ± 4.7	−25.9 ± 3.9	n.s.
Circumferential strain rate (SAXB) (%)	−24.4 ± 3.2	−24.0 ± 4.4	n.s.
Global longitudinal strain rate (%)	−20.1 ± 2.3	−20.7 ± 2.5	n.s.
Longitudinal strain rate (AP4) (%)	−19.9 ± 2.5	−20.2 ± 3.0	n.s.
Longitudinal strain rate (AP2) (%)	−20.6 ± 3.2	−20.9 ± 3.1	n.s.
Longitudinal strain rate (AP3) (%)	−20.3 ± 2.3	−21.6 ± 2.8	n.s.
Stress			
Global circumferential strain rate (%)	−24.2 ± 3.9	−23.8 ± 4.1	n.s.
Circumferential strain rate (SAXM) (%)	−24.6 ± 4.0	−23.9 ± 4.6	n.s.
Circumferential strain rate (SAXB) (%)	−23.3 ± 4.1	−23.3 ± 4.3	n.s.
Global longitudinal strain rate (%)	−21.6 ± 2.9	−21.0 ± 2.7	n.s.
Longitudinal strain rate (AP4) (%)	−21.6 ± 3.1	−21.0 ± 2.3	n.s.
Longitudinal strain rate (AP2) (%)	−21.8 ± 3.7	−21.2 ± 0.5	n.s.
Longitudinal strain rate (AP3) (%)	−21.7 ± 3.7	−21.7 ± 4.4	n.s.

SAXM: parasternal short axis view at the papillary muscle plane, SAXB: parasternal short axis view at the mitral valve plane, AP4: apical four-chamber view, AP2: apical two-chamber view, and AP3: apical three-chamber view; *p* values calculated with Man-Whitney *U* test, level of significance = 0.05.
